# The Long-Acting D3 Partial Agonist MC-25-41 Attenuates Motivation for Cocaine in Sprague-Dawley Rats

**DOI:** 10.3390/biom10071076

**Published:** 2020-07-18

**Authors:** Gregory L. Powell, Mark D. Namba, Annika Vannan, John Paul Bonadonna, Andrew Carlson, Rachel Mendoza, Peng-Jen Chen, Robert R. Luetdke, Benjamin E. Blass, Janet L. Neisewander

**Affiliations:** 1 School of Life Sciences, Arizona State University, PO Box 874501, Tempe, AZ 85287-4501, USA; gregory.powell.1@asu.edu (G.L.P.); mnamba@asu.edu (M.D.N.); avannan@asu.edu (A.V.); jpbonad@gmail.com (J.P.B.); carlson.andrew.k@gmail.com (A.C.); rachelmendoza@gwmail.gwu.edu (R.M.); 2 Department of Pharmaceutical Sciences, Moulder Center for Drug Discovery Research, Temple University School of Pharmacy, Philadelphia, PA 19140, USA; peng-jen.chen@temple.edu (P.-J.C.); tud12939@temple.edu (B.E.B.); 3 Department of Pharmacology and Neuroscience, Institute for Healthy Aging, University of North Texas Health Science Center, Fort Worth, TX 76107, USA; Robert.Luedtke@unthsc.edu

**Keywords:** dopamine D3 receptors, cocaine, motivation, behavioral economics, reinforcement, self-administration

## Abstract

The dopamine D3 receptor is a prime target for developing treatments for cocaine use disorders (CUDs). In this study, we conducted a pre-clinical investigation of the therapeutic potential of a long-acting, D3 receptor partial agonist, MC-25-41. Male rats were pre-treated with MC-25-41 (vehicle, 1.0, 3.0, 5.6, or 10 mg/kg, intraperitoneal (IP)) five minutes prior to tests of cocaine or sucrose intake on either a progressive ratio schedule of reinforcement or a variable interval 60 s multiple schedule consisting of 4, 15-min components with sucrose or cocaine available in alternating components. A separate cohort of rats was tested on a within-session, dose-reduction procedure to determine the effects of MC-25-41 on demand for cocaine using a behavioral economics analysis. Finally, rats were tested for effects of MC-25-41 on spontaneous and cocaine-induced locomotion. MC-25-41 failed to alter locomotion, but reduced reinforcement rates for both cocaine and sucrose on the low-effort, multiple schedule. However, on the higher-effort, progressive ratio schedule of cocaine reinforcement, MC-25-41 reduced infusions, and active lever presses at doses that did not alter sucrose intake. The behavioral economics analysis showed that MC-25-41 also increased cocaine demand elasticity compared to vehicle, indicating a reduction in consumption as price increases. Together, these results suggest that similar to other D3-selective antagonists and partial agonists, MC-25-41 reduces motivation for cocaine under conditions of high cost but has the added advantage of a long half-life (>10 h). These findings suggest that MC-25-41 may be a suitable pre-clinical lead compound for development of medications to treat CUDs.

## 1. Introduction

Development of novel therapies for the treatment of cocaine use disorders (CUDs) remains a high priority given that the number of cocaine-related deaths is increasing in parallel with the opioid crisis underway in the United States since the 2010s [1,2]. Investigation of human cocaine-overdose victims has revealed increased expression of dopamine D3 receptors (D3Rs) within the nucleus accumbens [3], a critical region of the neurocircuitry for CUDs [4,5]. Similarly, pre-clinical research has shown up-regulation of D3R binding under conditions of high motivation for cocaine (reviewed by [6]). New drugs targeting the D3R represent an exciting avenue for treating CUDs as well as opiate use disorders [7]. Indeed, D3R compounds are among the “ten most wanted pharmacological mechanisms” intended for rapid development by the National Institute of Drug Abuse’s Division of Therapeutics and Medical Consequences [8].

As thoroughly reviewed by Sokoloff and Le Foll [9], D3R drugs reduce cocaine-motivated behaviors on high- but not low-effort schedules of reinforcement. Dopamine D3R partial agonists and antagonists are generally ineffective in altering cocaine intake on low fixed ratio schedules requiring 5 or fewer responses [10,11,12,13,14]. Similarly, we have found that cocaine intake on a low-effort variable interval (VI) 60 s schedule of reinforcement is only affected at doses of D3R partial agonists and antagonists that also affect sucrose reinforcement and cocaine-induced locomotor activity [11]. In contrast, on high-effort progressive ratio (PR) schedules that require progressively more responses for reinforcement, D3R partial agonists and antagonists selectively decrease cocaine intake [11,15]. Meanwhile, D3R drugs have also been shown to reduce cue-induced reinstatement of cocaine-seeking behavior, suggesting potential utility in the reduction of craving elicited by cocaine-associated cues [11,16]. Together, these data suggest a consistent profile for D3R drugs as potential anti-craving treatments for CUDs.

Behavioral economics analysis is another method of examining pharmacological treatment effects on motivation for drug. This approach has gained traction recently because of parallels that can be drawn between animal and human research based on parameter estimates of both demand intensity and demand elasticity [17,18]. Demand intensity is measured as consumption of drug at high doses/low unit prices (*Q*_0_), i.e., consumption when the drug is virtually free. Elasticity of demand is typically measured as the rate of change in consumption across unit price (α), though other measures such as *P_max_* (the unit price of maximal responding) and *O_max_* (the maximal rate of responding) are common as well. These measures of elasticity assess willingness to continue working for drug as price increases and are thought to reflect motivation for the commodity. Previously, various doses of the D2-like receptor antagonist haloperidol were shown to reduce *P_max_* and increase cocaine consumption at high doses of cocaine [19], suggesting that the behavioral economics paradigm is capable of detecting changes in demand due to pharmacological treatments. Recent studies provide further support for the utility of behavioral economics analysis of pharmacotherapeutic treatments on the demand for opioids [20,21] and methamphetamine [18,22].

**Figure 1:**
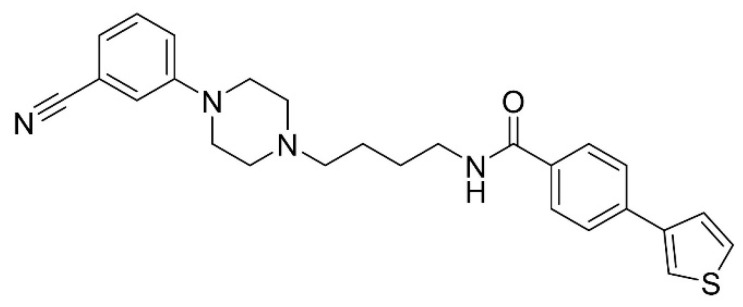
Structure of MC-25-41.

Here, we report the effects of the D3R-selective partial agonist, MC-25-41, on reinforcer intake in male rats tested under either low-effort or increasing-effort schedules of cocaine and/or sucrose reinforcement. MC-25-41 demonstrates high affinity for the D3R (K_i_ = 0.50 nM), selectivity for the D3R over the D2R (1486-fold), a prolonged half-life (>60 m in human and rat liver microsome assays), and low efficacy (19.4% maximum activity) in the forskolin-dependent adenylyl cyclase inhibition assay ([23]; referred to as “13r”; Figure 1). The low-effort reinforcement schedule used in the present study was a VI 60 s multiple schedule of cocaine and sucrose reinforcement. The increasing-effort schedules included a PR schedule of either cocaine or sucrose reinforcement and a within-session, cocaine dose-reduction procedure [24]. Rats were also tested for the effects of MC-25-41 on spontaneous and cocaine-induced locomotor activity.

## 2. Methods

### 2.1. Animals

Adult male Sprague–Dawley rats (Charles River, Hollister, CA, USA) weighing 200–225 g upon arrival were maintained on a 14:10 reverse light: dark cycle. Rats were handled daily for at least 5 days prior to surgical implantation of catheters. Water was available ad libitum during all experiments. Access to food was restricted to maintain rats at 85% of their estimated free-feeding weight during recovery from catheter implant surgery and during initial cocaine self-administration training sessions. All tests of MC-25-41 effects on cocaine or sucrose reinforcement were conducted when animals had ad libitum access to food in the home cage except where otherwise noted. All procedures were approved by the Arizona State University Institutional Animal Care and Use Committee and followed NIH guidelines.

### 2.2. Surgery

For self-administration studies, animals underwent jugular vein catheter implantation as previously described [25]. Briefly, a catheter was implanted into the rat’s jugular vein and tunneled subcutaneously along the neck to an incision on top of the head. Dental acrylic was used to secure the catheter’s metal cannula (22 gauge) end to the surface of the skull. Cefazolin (100 mg/mL, intravenous (IV)) antibiotic was delivered daily for 5 days following surgery and saline with heparin (70 U/mL, IV) was administered daily throughout the experiments to maintain catheter patency. Catheter function was assessed with methohexital sodium (16.67 mg/mL, IV), which produces brief loss of muscle tone only when administered IV [26].

### 2.3. Drug Preparation

MC-25-41 was prepared according to the methods described in our previous publication [23] and dissolved in 20% *w*/*v* cyclodextrin and 3% *v*/*v* 1M hydrochloric acid and was delivered IP at a volume of 1 mL/kg. Cocaine hydrochloride (RTI International, Research Triangle Park, NC, USA) was dissolved in bacteriostatic saline and additionally filtered through 0.3 μm filters when administered IV. For cocaine-induced locomotion, cocaine hydrochloride was administered IP at volume of 1 mL/kg.

### 2.4. Locomotion

Experimentally naïve animals were tested for effects of MC-25-41 on cocaine-induced and/or spontaneous locomotor activity following similar procedures to those previously described [11]. All animals were randomly assigned to groups that received one of three doses of MC-25-41 (1.0, 3.0, or 5.6 mg/kg IP; *n* = 8–12/dose) prior to tests of drug effects and they also received vehicle prior to separate control tests, with the order of drug and vehicle tests counterbalanced. To assess MC-25-41 effects on spontaneous locomotion, animals received their injection of vehicle or MC-25-41 and 15 min later they were placed into Plexiglas assessment chambers (44 × 24 × 20 cm high) where distance travelled (m) was recorded by a computer-automated video tracking system (Clever Systems, Reston, VA, USA) for 1 h. To assess MC-25-41 effects on cocaine-induced locomotion, animals were initially placed into the chambers for a 1-h habituation session without drug pre-treatment. After habituation, they received their injection of vehicle or MC-25-41 and were placed into their home cage. Between 5–10 min later, animals received an injection of cocaine hydrochloride (15 mg/kg IP) and were immediately returned to the testing chambers to track cocaine-induced locomotor activity for 1 h.

### 2.5. Progressive Ratio Schedule

Animals were food-restricted (18 g/day) to facilitate acquisition of cocaine self-administration. Training sessions took place 5–6 days/week in operant conditioning chambers (30 × 24 × 21 cm; Med Associates, Inc., St. Albans, VT, USA) equipped with two retractable levers, of which one was designated the active lever and resulted in reinforcement when pressed, and the other inactive lever produced no consequences when pressed. Training began with 2-h sessions during which cocaine was initially available on a fixed ratio 1 (FR 1) schedule of reinforcement, where a single active lever press activated a light cue above the active lever and a tone cue (500 Hz) followed 1 s later by delivery of a 0.75 mg/kg IV cocaine infusion (0.1 mL volume) over 6 s. All these stimuli (pump, cue light, tone) were then inactivated simultaneously and a house light was illuminated to signal a 20-s timeout period during which lever responses produced no consequences. Within-session, the schedule increased to variable ratio (VR, in which an average number of lever presses is needed for each reinforcer) 2, 3, and 5 sequentially. The criterion for advancement of the schedule was reached when an animal received 5 infusions in any given hour. Between sessions, the starting schedule (FR 1, or VR 2, 3 or 5) increased when an animal ended the previous 3 sessions on a higher schedule than the starting one. The training dose of cocaine was selected because it supports acquisition in most rats while avoiding toxicity that can occur with higher doses.

Once animals had stabilized on a VR 5 starting schedule and their infusions varied ≤15% across three consecutive sessions, all animals were switched to ad libitum food access in their home cage and training began on a PR schedule of 0.375 mg/kg, IV cocaine reinforcement with the session length increased to 3 h. On the PR schedule of reinforcement, the number of active lever responses required for successive reinforcers increased according to the formula 5*e*^0.2*n*^ − 5, rounded to the nearest integer, where *n* is the *n*th reinforcer in a session [27]. The cocaine dose was reduced for this phase of the experiment to capture break points, operationally defined as no reinforcer received for 1 h or no levers pressed for 30 min. Most rats reach break points within the 3-h session length at this lower cocaine dose whereas it takes much longer for them to reach break point at the higher training dose. During the PR training/testing phase, food was given ad libitum to rats in their home cages. PR sessions ended for a given rat after reaching a breakpoint or after 3 h had elapsed, whichever occurred first. Animals were tested on separate days for effects of each dose of MC-25-41 (vehicle, 3.0, 5.6, and 10 mg/kg, IP) after reaching a criterion of less than 15% variability in infusions across 3 days during sessions intervening test days. On test days, animals were pre-treated with MC-25-41 5 min before placement into the operant conditioning chamber. Doses were counterbalanced for each animal.

### 2.6. Multiple Schedule of Sucrose and Cocaine Reinforcement

Self-administration tests took place in the same chambers as described above using a procedure similar to that used previously [28]. Prior to training, animals were food-restricted (18 g/day) to facilitate acquisition. Food was gradually increased as self-administration progressed. Animals initially underwent training for sucrose (45 mg pellets; Bio-Serv, Frenchtown, NJ, USA), then cocaine (0.75 mg/kg, IV), reinforcement in which the location of the active lever for each reinforcer was counterbalanced between animals (i.e., for half of the rats the left lever was the active lever for sucrose and the right lever was the active lever for cocaine, and vice versa for the other half). The active lever was signaled by a cue light above the lever, which remained illuminated throughout except during the 20 s time out periods that followed reinforcer delivery. All training sessions were 2 h and occurred daily 6 days/week regardless of reinforcer. For sucrose training sessions, animals progressed within-session from FR 1 to VI 10, 30, and 60, with the schedule increasing each time animals received 5 reinforcers within 40 min. For VI schedules, an active lever press confers 1 reinforcer after a given time block that varies but averages to the time specified in the schedule (e.g., in a VI 60 s schedule, animals receive 1 reinforcer upon pressing the active lever once after an average of 60 s has elapsed). After concluding on a VI 60 s schedule for 3 consecutive sessions, the starting schedule was changed to VI 60 s. Once animals acquired sucrose self-administration, defined as at least 14 reinforcers received during each of 3 consecutive sessions, cocaine reinforcement (0.75 mg/kg, IV) training began in the same operant conditioning chambers where the previously inactive lever was the active lever, signaled by a cue light as described for the sucrose reinforcement session. Training schedules progressed as with sucrose (FR 1, then VI 10 s, 30 s and 60 s) until at least 14 cocaine reinforcers were received during each of 3 consecutive sessions.

After rats met the acquisition criterion for both sucrose and cocaine reinforcement schedules, multiple schedule sessions began. During this time, food was given ad libitum in the home cage. Sessions consisted of eight 15-min components beginning with a sucrose component and alternating between cocaine and sucrose as the available reinforcer thereafter. The components were signaled by the cue light above the designated active lever for the respective reinforcers as done during the initial training sessions. A 1-min timeout occurred between successive components, during which both levers were retracted and neither cue light was illuminated. Stability criterion to qualify for testing was defined as 15% or less variability in the reinforcers obtained during the previous 3 session on the VI 60 s schedule. During the testing phase, animals were injected with vehicle or MC-25-41 (3.0, 5.6, or 10 mg/kg, IP) 5 min before the session. Testing sessions were the same as training sessions except that they consisted of only 4 alternating 15-min components rather than 8. Dose order across tests was counterbalanced, with animals receiving each dose once. At least 3 sessions without MC-25-41 pre-treatment took place between testing sessions to re-achieve stability criteria.

### 2.7. Within-Session Cocaine Self-Administration Dose-Response Function

The effects of MC-25-41 on cocaine demand were assessed using a within-session dose-reduction procedure. Animals remained on food restriction throughout testing on the within-session cocaine self-administration procedures to maintain 90% free-feeding body weight. Rats acquired self-administration (0.75 mg/kg/0.1 mL) on an FR1 schedule of reinforcement as described above for the PR experiments. A minimum of seven days with at least 10 infusions per 2-h session and stable reinforcement rates of less than 15% variability in infusions across three consecutive sessions were necessary for rats to transition to the dose-reduction procedure. During dose-reduction sessions, 9 different doses of cocaine (1, 0.5623, 0.3162, 0.1778, 0.1, 0.0562, 0.0316, 0.0178, and 0.01 mg/kg) were available response-contingently with each dose available for 10 min in descending order of doses. Rats received an additional 10-min block at the highest dose to begin the session, though this block was excluded from data analysis as rats often “front-load” consumption at the beginning of a session [19,29]. Dose was modulated by decreasing the duration of the infusion delivered, from a maximum of 8 s down to 0.08 s. The light and tone cues were reduced in conjunction with the reduction in dose. Stable performance was determined for each individual rat as less than 25% variability in demand elasticity (α) across 3 sessions. On test days, vehicle or MC-25-41 (10 mg/kg, IP) was administered 5-min prior to the session. A minimum of 3 additional dose-reduction sessions without pre-treatment was given between test days.

### 2.8. Statistical Analysis

Statistical analyses were performed in SPSS 25 (IBM) or Prism 8.1 (GraphPad). For locomotor activity, two-way mixed model analysis of variance (ANOVA) were used to assess the effect of dose of MC-25-41 (vehicle, 1.0, 3.0, 5.6, or 10 mg/kg, IP) and time (10-min bin) on distance travelled. For the PR experiment, a one-way repeated measures ANOVA assessed the effect of dose (vehicle, 1.0, 3.0, 5.6, or 10 mg/kg) on cocaine intake and lever presses as a percent of baseline (the last 10-min bin of the habituation session). For multiple schedule data, 2-way repeated measures ANOVAs were used to assess the number of reinforcers received as well as active and inactive lever presses by dose (vehicle, 3.0, 5.6, or 10 mg/kg) and reinforcer type (sucrose or cocaine). For repeated measures ANOVAs that violated sphericity, Greenhouse–Geisser corrections were performed. Significant effects were further analyzed by post hoc Dunnett tests, Tukey’s *t*-tests, or Bonferroni t-tests to correct for multiple comparisons. Behavioral economics analysis was used to analyze cocaine demand from data collected during the within-session dose-reduction procedure. Cocaine consumption was determined from the number of infusions earned in each 10-min block multiplied by the dose administered. The exponentiated demand equation [30]:

(1) $$Q=Q_{0}*10^{k\left(e^{-\alpha Q_{0}C}-1\right)}$$

was fit to group data via the nonlinear mixed effects modeling package “nlme” in R [31,32], where *Q* is consumption; *Q*_0_ is consumption at a theoretical unit price of zero; *k* is a global constant; α is demand elasticity; and *C* is unit price. Unit price is defined as the ratio requirement to receive an infusion (e.g., 1 for the studies discussed here) divided by the dose of drug administered. The models defined *Q*_0_ and α as free parameters, *k* as a global constant (1.249), unit price as a fixed, continuous within-subjects factor, treatment as a fixed, between-subjects factor, and subject as a random factor. *P_max_*, the price at which maximal responding occurs, was calculated using the following equation [33]:

(2) $$P_{max}=\frac{0.084k+0.65}{Q_{0}*\alpha *k^{1.5}}$$

with *Q*_0_, *α*, and *k* representing the values determined from the nonlinear mixed effects modeling described above.

An alpha level of 0.05 was set for all experiments and all descriptive statistics are reported as the mean ± standard error of the mean (SEM).

## 3. Results

### 3.1. Spontaneous and Cocaine-Induced Locomotion

**Figure 2:**
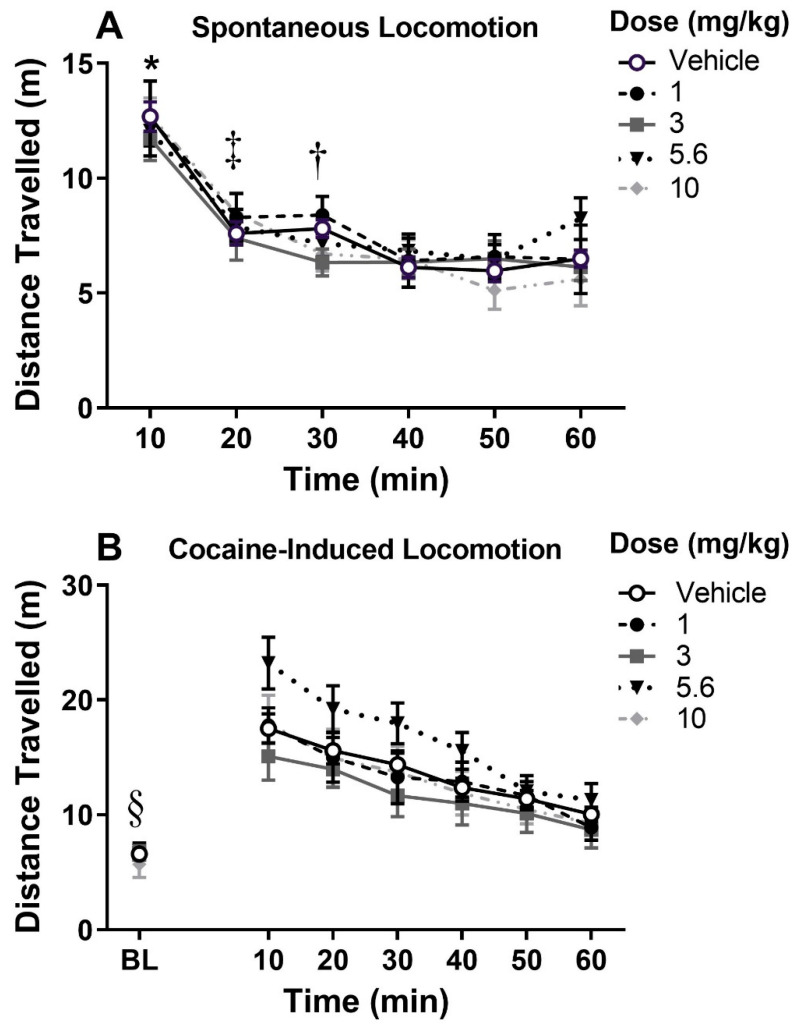
Spontaneous (**A**) and cocaine-induced locomotion (**B**) in rats after vehicle or an assigned dose of MC-25-41 (1.0, 3.0, 5.6, or 10 mg/kg, IP; *n* = 8–12/dose). Data are the mean ± standard error of the mean (SEM). * indicates difference from all other time bins with post hoc Tukey’s test. ‡ indicates difference from 40, 50, and 60 min time bins with post hoc Tukey’s test. † indicates difference from 40, 50 time bins with post hoc Tukey’s test. § indicates baseline difference from all other time points with post hoc Dunnett’s test.

For spontaneous locomotion (*n* = 8–12/dose; Figure 2A), the ANOVA indicated a main effect of time (F_3.10,117.83_ = 91.49, *p* < 0.05), but no main effect of dose nor interaction. The effect of time was due to the decrease in locomotion across time as rats habituated to the environment. Tukey’s multiple comparison tests indicated that the first 10-min bin was significantly different from every other bin (*p* < 0.0001); the 20-min bin was significantly different from the 40-, 50-, and 60-min bins (*p* < 0.0001, *p* < 0.0001, and *p* < 0.05, respectively); and the 30-min bin was significantly different from the 40- and 50-min bins (*p* < 0.0005 and *p* < 0.0001, respectively). The ANOVA of cocaine-induced locomotion (*n* = 8–12/dose; Figure 2B) also revealed a significant main effect of time (F_2.06,78.22_ = 48.65, *p* < 0.05), but no main effect of dose nor interaction. The main effect of time was due to a cocaine-induced increase in locomotion from baseline to the first 10-min block (Dunnett’s multiple comparison test, q_83_ = 13.67, *p* < 0.05), demonstrating cocaine-induced locomotion regardless of dose of MC-25-41. Thus, MC-25-41 at the doses tested had no effect on spontaneous or cocaine-induced locomotion.

### 3.2. Progressive Ratio Schedule of Cocaine or Sucrose Reinforcement

**Figure 3:**
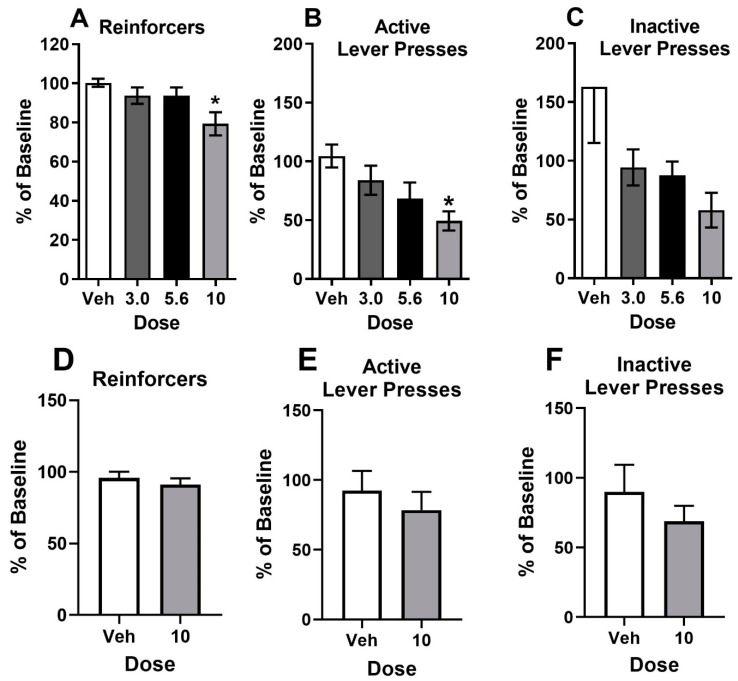
Effects of MC-25-41 (Vehicle, 3.0, 5.6, and 10 mg/kg) on behavioral measures under a progressive ratio (PR) schedule of reinforcement. Rats (*n* = 7) tested on the PR schedule of cocaine reinforcement (**A**–**C**) were tested on each of the four doses on separate test days. Rats (*n* = 17) tested on a PR schedule of sucrose reinforcement (**D**–**F**) were each tested on two doses of MC-25-41 (Vehicle or 10 mg/kg, IP) on separate test days. Test day measures are shown as a percentage of baseline values ± SEM. * indicates difference from vehicle with post hoc Dunnett’s test.

MC-25-41 dose-dependently reduced cocaine intake and active lever responses on the PR schedule of reinforcement (*n* = 7). Behavioral measures on the PR schedule changed across the sessions between tests but were stable prior to each test. Due to this drift in baselines, infusions and lever presses for a given test day were normalized to rates of these measures the day prior and were analyzed as a percentage of this baseline. For cocaine self-administration, ANOVA of infusions revealed a main effect of dose (F_3,15_ = 9.40, *p* < 0.05; Figure 3A). Post hoc Dunnett’s test indicated a significant difference between the 10 mg/kg dose and vehicle (q_5_ = 5.135, *p* < 0.05). For active lever presses, ANOVA indicated a main effect of dose (F_3,15_ = 4.42, *p* < 0.05; Figure 3B). Vehicle was again significantly different from 10 mg/kg, IP dose of MC-25-41 (q_5_ = 3.50, *p* < 0.05, Dunnett’s test). There were no significant effects for inactive lever presses (Figure 3C). For sucrose reinforcement (*n* = 17), the 10 mg/kg, IP dose of MC-25-41 had no effect on the number of reinforcers obtained (Figure 3D), active lever presses (Figure 3E), or inactive lever presses (Figure 3F) expressed as a percentage of baseline, even though this dose had reduced cocaine intake under the same PR schedule of reinforcement.

### 3.3. Multiple Schedule of Sucrose and Cocaine Reinforcement

**Figure 4:**
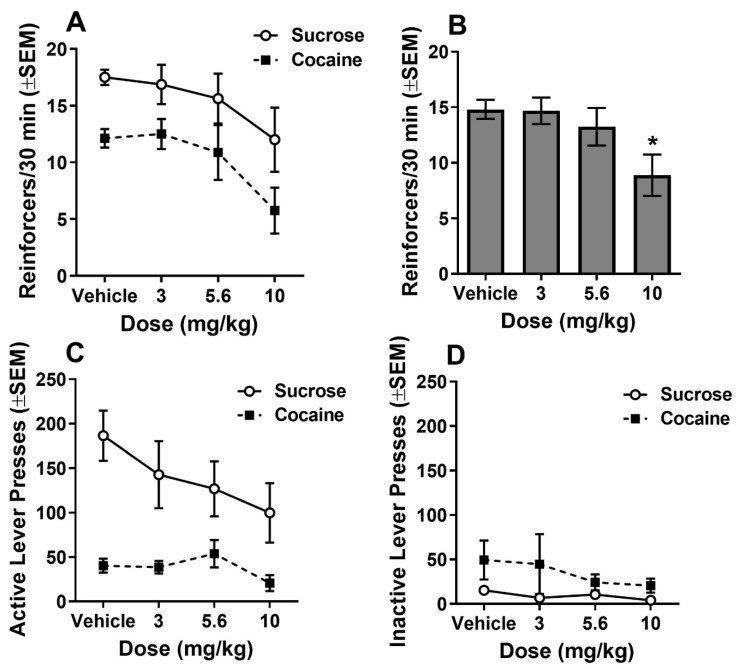
Multiple schedule of sucrose and cocaine reinforcement. Animals (*n* = 8) were each tested on each of four doses of MC-25-41 (Vehicle, 3.0, 5.6, and 10 mg/kg) on a multiple VI60 s schedule in which 15-min schedule components alternated between sucrose (45 mg pellet) and cocaine (0.75 mg/kg, IV) availability for 1 hr. Shown are the individual reinforcers earned (**A**), reinforcers earned collapsed across reinforcer type (**B**), active lever presses (**C**), and inactive lever presses (**D**). Reinforcer and response rates were higher under the sucrose components compared to the cocaine components (main effect of reinforcer). Data points are the mean ± standard error of the mean (SEM). * indicates a significant difference from vehicle.

For reinforcers obtained under the multiple VI 60s schedule of cocaine and sucrose reinforcement (*n* = 8; Figure 4A), ANOVA indicated a main effect of dose (F_2.05,28.66_ = 5.20, *p* < 0.05). Subsequent Dunnett’s tests indicated a reduction in reinforcers earned at the 10 mg/kg, IP dose of MC-25-41 compared to vehicle (q_15_ = 3.20, *p* < 0.05) regardless of reinforcer. A significant main effect of reinforcer (F_1,14_ = 9.98, *p* < 0.05) was also observed, indicating that the rats obtained more sucrose reinforcers than cocaine reinforcers across all doses of MC-25-41 tested. There was no dose by reinforcer interaction. ANOVA of active lever presses revealed a significant main effect of reinforcer (F_1,14_ = 20.58, *p* < 0.05) but found no effect of dose nor dose by reinforcer interaction (Figure 4B). These results indicate that rats pressed the active lever more during the sucrose components than during the cocaine components of the schedule. For inactive lever presses, there were no significant main effects or interactions (Figure 4C).

### 3.4. Behavioral Economics Analysis of Cocaine Self-Administration

**Figure 5:**
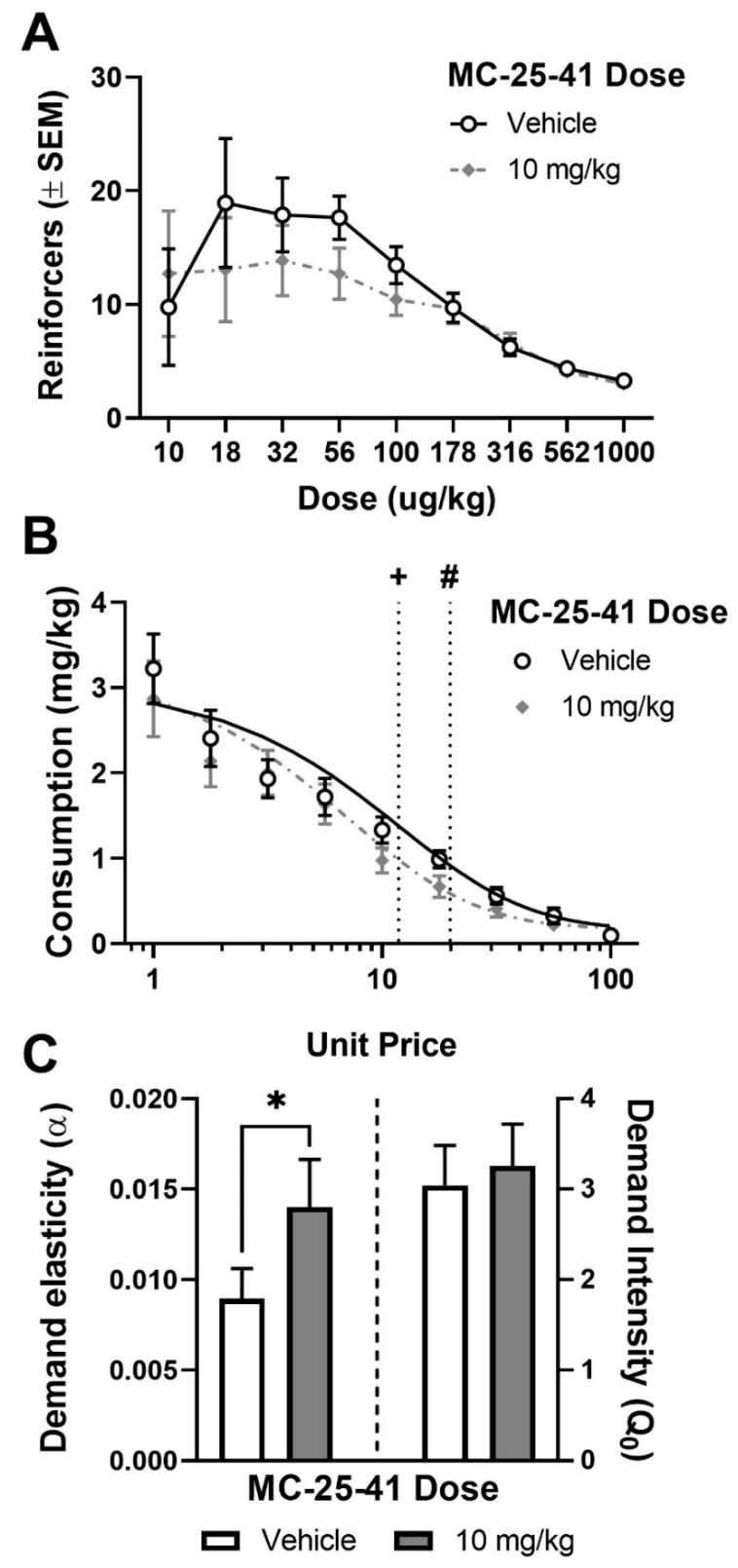
Cocaine demand assessment using a within-session, dose-reduction procedure, and behavioral economic analysis. Animals (*n* = 17) were each tested on two doses of MC-25-41 (Vehicle and 10 mg/kg) prior to access to 9 different doses of cocaine, each available for 10 min in descending order. Shown are the dose-effect functions (**A**), demand functions (**B**), and parameter estimates (**C**). +, P_max_ value after MC-25-41 treatment. **#**, P_max_ value after vehicle treatment. Data points are the mean ± standard error of the mean (SEM). * indicates a difference from vehicle.

The traditional dose-response function generated using the within-session, dose-reduction procedure after treatment with vehicle or 10 mg/kg, IP MC-25-41 is shown in Figure 5A (*n* = 17). ANOVA of reinforcers indicated a significant main effect of cocaine dose (F_8,261_ = 5.68, *p* < 0.05), but no main effect of treatment and no interaction between cocaine dose and treatment. Multiplying the number of infusions by the dose provided when the infusion was earned produces a measure of consumption for each unit price. Demand functions were fit to consumption data (Figure 5B) and parameter estimates for demand intensity (*Q*_0_) and demand elasticity (α) were determined (Figure 5C). Treatment with 10 mg/kg, IP MC-25-41 increased demand elasticity (α) compared to vehicle (F_1,258_ = 8.91, *p* < 0.05), indicating a reduction in consumption of cocaine as unit price increased. However, no difference in demand intensity was observed between drug and vehicle tests. Using the Hursh and Roma equation described in the Methods to calculate *P_max_*, the price at which maximal responding occurs, from the demand parameters shown in Figure 5C yielded a value of 19.82 for vehicle and 11.80 for MC-25-41. Lower *P_max_* and higher α suggests that MC-25-41 decreased motivation for cocaine.

## 4. Discussion

MC-25-41, a long-acting dopamine D3R partial agonist, reduced breakpoints for cocaine reinforcement on the high-effort PR schedule (Figure 3) and increased demand elasticity without altering demand intensity on the within-session dose-reduction procedure (Figure 5). On the low-effort VI schedule used for comparing cocaine and sucrose reinforcement, MC-25-41 decreased the reinforcement rate for both sucrose and cocaine at the highest dose tested (10 mg/kg, IP) (Figure 4). As MC-25-41 did not alter spontaneous or cocaine-induced locomotor activity (Figure 2) it is unlikely that any of these effects were due to a decrease in locomotion.

Demand elasticity is known as an inverse measure of motivation because as elasticity increases, motivation for drug is thought to decrease [20]. Other studies have shown that pharmacological treatments increase elasticity for cocaine [18,34], methamphetamine [22], and remifentanil [21] and these results are interpreted as a treatment-induced decrease in motivation for drug. Similarly, these treatments have been shown to reduce cue-induced drug-seeking for cocaine, methamphetamine, and remifentanil, suggesting that α may be a useful index of drug motivation. In this study, MC-25-41 increased demand elasticity but failed to reduce cocaine demand intensity (*Q*_0_), the parameter associated with the reinforcing value of cocaine. Our previously tested D3R partial agonists and antagonists also failed to reduce consumption of cocaine at a unit dose of 0.75 mg/kg/infusion or greater on a VI 60 s schedule of reinforcement [11,28,35], suggesting cocaine reinforcement is not affected. Together, these results are consistent with the hypothesis that D3R partial agonists and antagonists alter motivation for cocaine, but not cocaine reinforcement [11,36,37,38,39,40].

Our finding that MC-25-41 reduced cocaine intake when rats were tested on the higher-effort PR schedule is consistent with previous studies with other D3R antagonists and partial agonists. We have previously reported, for example, that a D3R-selective partial agonist phenylpiperazine, LS-3-134, successfully reduces cocaine intake on a PR schedule [11]. Several other studies have shown similar effects of D3R antagonists and partial agonists when animals are tested on a PR schedule for cocaine reinforcement [13,15,16,41]. Additionally, when tested on a PR schedule for methamphetamine, D3R partial agonists and antagonists reduce drug intake [42]. Therefore, these effects on higher-effort schedules are further indicative of the role of D3 receptors in motivation for drugs and align with the interpretations suggested above from the behavioral economic analysis.

Upward vertical shifts in the cocaine dose-response function are thought to represent an enhancement of the reinforcing effects of drugs [43,44]. Alternatively, upward vertical shifts may represent an increase in the hedonic set point (i.e., more drug is necessary to achieve the hedonic set point) [45], and thus downward vertical shifts would represent a decrease in the hedonic set point. An alternative hypothesis put forth by Egli et al. (2016; [46]) attributes vertical shifts in the dose-response function to changes in motivation. The analysis of the cocaine intake dose-effect function in the present study found no main effect of treatment on the dose-response function nor any interaction between treatment and cocaine dose indicative of a vertical downward shift of the dose-effect function. Caution must be taken in interpreting this negative finding especially since the variance at a given cocaine dose in the within-session dose-reduction procedure may obscure detecting changes in the dose-response function. By contrast, the behavioral economics mathematical modeling parameters are derived from the entire dose-effect function, and therefore, detected changes that were evidently missed by traditional ANOVA. The lack of change in demand intensity (Q_0_) in the present study suggests that cocaine reinforcement was unaltered whereas the MC-25-41-induced increase in demand elasticity suggests a decrease in motivation for cocaine. The latter interpretation is further reinforced by the MC-25-41-induced reduction in infusions on the PR schedule of cocaine reinforcement.

The general reduction in cocaine and sucrose reinforcement observed when the high dose of MC-25-41 (10 mg/kg, IP) was administered prior to the VI 60 s multiple schedule (Figure 4) was unexpected. In previous studies, a general reduction in reinforcement following treatment with D3R antagonists or partial agonists was observed at doses that also alter locomotor activity [12,13,15,16,28,35,47,48,49,50,51], suggesting behavioral disruption rather than an effect on reinforcement, perhaps through an effect at receptors other than D3Rs. Given that MC-25-41 had no effect on spontaneous or cocaine-induced locomotion in the present study at any dose tested, including 10 mg/kg, IP, the MC-25-41-induced reduction in cocaine and sucrose intake at the high dose of MC-25-41 may be due to a D3R-induced decrease in reinforcement or motivation in general. However, the lack of effect of MC-25-41 on sucrose intake on the PR schedule mitigates a general decrease in motivation. This suggests that the MC-25-41-induced decrease in cocaine and sucrose intake under the low-effort VI 60 s schedule is most likely due to a decrease in the efficacy of these reinforcers, although it remains to be determined whether this is due to an action at dopamine D3Rs.

The pharmacokinetic profile and in vivo pharmacology of MC-25-41 suggest that it may be a useful pre-clinical lead compound [23]. MC-25-41 has sub-nanomolar affinity for D3Rs (K_i_ = 0.50 nM), high selectivity for D3 over D2 receptors (1486 fold), and a half-life in male Sprague–Dawley rats of over 10 h [23]. Beyond the selectivity for D3R over D2R, MC-25-41 has >20,000-fold selectivity for D3R over D1R or D5R and >1600-fold selectivity for D3R over D4R. Furthermore, MC-25-41 has >20,000-fold selectivity for a list of other targets potentially involved with substance use disorders, including adrenergic receptors (α_1A/1B/1D/2A/2B/2C_ and β_1/2/3_), histamine receptors (H_1/2/3/4_), muscarinic cholinoreceptors (M_1/2/3/4/5_), opioid receptors (δ, κ, and µ), and the dopamine and norepinephrine transporters. Therefore, the effects we observed are most likely D3R-mediated. MC-25-41 reportedly does bind to the 5-HT_2C_ receptor (K_i_ = 20 nM), a receptor known to have similar effects as D3R compounds in reducing cocaine self-administration, cocaine-seeking behaviors, and locomotor activity [52,53]. This high level of selectivity minimizes the risk of off-target toxicities associated with the aforementioned targets.

D3R modulators may have utility as treatments administered during abstinence from drug use, as they may reduce drug craving without producing the side effects caused by drugs targeting D2 receptors. Beyond the potential of D3R modulators to reduce cocaine-motivated behaviors, they may also have utility in treating opioid and nicotine use disorders [54,55,56,57,58]. Importantly, research on D3R modulators to date has primarily used male rats. Additional studies that employ female rats are needed as sex differences in dopamine systems are documented and will be crucial to investigate for the advancement of D3R treatment strategies [59,60,61,62,63]. Also given that other D3R partial agonists reduce cocaine-seeking behavior in the reinstatement model [11,36,37,38,39,40], future research using this model would further support our observed MC-25-41-induced changes in behavioral economic parameters reflecting a reduction in motivation for cocaine.

## 5. Conclusions

MC-25-41, a highly selective D3R partial agonist with a long half-life, reduces cocaine-motivated behaviors on high and low-effort schedules and may have an impact on reward efficacy. Furthermore, the analysis of MC-25-41 pharmacological effects using a behavioral economics framework is a novel approach for evaluating pharmacotherapeutics targeting the dopamine system. This approach identified effects on motivation, specifically an increase in demand elasticity, missed by traditional analysis of cocaine self-administration dose-response data. These findings together with the promising pharmacokinetic properties of MC-25-41 suggest it may be a useful pre-clinical candidate for the treatment of CUD.
